# Is the age at surgery in Crohn’s disease clinically relevant? Differences and peculiarities: a wide single centre experience after long-term follow-up

**DOI:** 10.1007/s00423-022-02613-6

**Published:** 2022-07-25

**Authors:** Cristina Luceri, Gabriele Dragoni, Daniela Zambonin, Benedetta Pesi, Edda Russo, Stefano Scaringi, Ferdinando Ficari, Fabio Cianchi, Francesco Giudici

**Affiliations:** 1 NEUROFARBA Department, Pharmacology and Toxicology Section, University of Florence, Florence, Italy; 2 Department of Experimental and Clinical Biomedical Sciences “Mario Serio”, University of Florence, Florence, Italy; 3 Department of Experimental and Clinical Medicine, University of Florence, Largo Brambilla 3, 50134 Florence, Italy

**Keywords:** Crohn’s disease, Age, Surgery, Outcome, Recurrence, Follow-up

## Abstract

**Purpose:**

The Montreal classification for Crohn’s disease includes “age at diagnosis” as a parameter but few is reported about the age at surgery. The aim of this study is to evaluate the short- and long-term differences in the postoperative surgical outcome and disease behaviour, according to the age at the first surgery.

**Methods:**

Patients consecutively operated for abdominal Crohn’s disease during the period 1986–2012 at our centre were systematically analysed according to their age at first surgery. In our retrospective cohort, the age at first surgery ranged from 13 to 83 years, and patients were arbitrarily divided into four groups: ≤ 19 (G1), 20–39 (G2), 40–59 (G3) and ≥ 60 (G4) years old.

**Results:**

In total, 1051 patients were included with a median follow-up time of 232 months. The four groups exhibited statistically significant differences in age at diagnosis, smoke habit, time between diagnosis and surgery, disease location and behaviour, history of perianal fistula or abscess, severe malnutrition requiring total parental nutrition before surgery, type of surgery, total length of resected bowel, median duration of hospitalization, incidence of abdominal recurrences and number of surgical recurrences. G1 displays an inverse linear trend with time in the severity of clinical characteristics when compared to G4 groups. On the contrary, the incidence of short-term complications, types of abdominal recurrence and presence of concomitant perianal disease did not vary among groups. In addition, at multivariate analysis, the age at surgery and the disease location were the only independent risk factors for abdominal surgical recurrence.

**Conclusion:**

Despite first surgery is extremely more frequent between 20 and 59 years, patients from G1 and G4 groups showed clinical differences and peculiarities when compared to the other age groups. The most indolent CD behaviour and occurrence of surgical recurrence was observed in patients having their first abdominal surgery in the elderly, while patients operated before the age of 19 experienced a more aggressive disease course.

## Introduction

In 2019, the Italian population aged over 65 was 23%. It has been estimated that, with the actual mean life expectancy of 81 years for males and 85 years for females, in 2045, 33.5% of the population will be older than 65 years, i.e. 20 million people [1].

Even in Europe, the rising incidence of Crohn’s disease (CD) added to the growth of an ageing population is contributing to an exponential increase of elderly CD patients [2]. Around 25–35% of CD patients are over the age of 60 years, and about 15% of these present a relatively late diagnosis [3, 4].

With advancing age, CD patients may present a higher number of comorbidities, whose association with an alternative clinical behaviour and a different surgical short- and long-term outcome has been poorly addressed.

It has been shown that the surgical risk of CD patients depends on the age at diagnosis. Interestingly, younger-onset CD exhibits a higher mortality than the general population, whereas this difference diminishes when comparing general population with elderly-onset CD [5].

Small experiences reported that elderly CD patients have a higher risk of surgery at diagnosis or shortly after diagnosis, but the long-term surgery rate is comparable to the adult-onset disease [2, 4, 6, 7]. However, the Montreal classification identifies only three CD groups according to the age of diagnosis (< 16, 17–39, ≥ 40 years) [8]. Therefore, many questions are still unsolved: is there a specific age at surgery when CD behaviour is different? Is CD operated for the first time after 60 years of age a different entity with different course and abdominal recurrence risk? Does the need of surgery before or after 60 years influence the surgical recurrence risk of CD patients? To clarify these issues, we here report an analysis of a very long observational period of a surgical CD cohort, divided into four groups by age at first CD surgery.

## Methods

We retrospectively reviewed a prospectively maintained database of all consecutive CD patients operated by the same surgical team at our Institution (Careggi University Hospital, Florence, Italy) during the period between January 1, 1986, and March 31, 2012. A local Ethical Committee approval was obtained before starting the inclusion. A multidisciplinary setting of at least one surgeon and one gastroenterologist experienced in inflammatory bowel diseases (IBD) discussed the decision to undergo surgery for all the enrolled patients.

Only patients with pathologically confirmed CD diagnosis that underwent concurrent intestinal resection and anastomosis/strictureplasty at first surgery were included into this study. We arbitrarily decided to stop the inclusion of patients at year 2012 to have a follow-up of at least 7 years in all cases. No patient at our centre had ever been excluded from surgical operation because of the advanced age. On January 2020 (follow-up end date), all patients were contacted by phone, and a survey designed by our clinician containing information on clinical symptoms, medications, radiological and endoscopic follow-up, surgical operations eventually performed was administered to collect their last follow-up clinical data; the ones not answering or refusing to participate were considered lost at follow-up and excluded from the analysis.

According to the age at first surgery, patients were arbitrarily divided in four groups: ≤ 19 (G1), 20–39 (G2), 40–59 (G3) and ≥ 60 (G4) years old, in order to have an homogeneous stratification of the cases representative of each age in our population. Clinical characteristics included were as follows: gender, smoke habit, age at diagnosis, family history of IBD (defined as the presence of at least a first degree relative affected with CD or ulcerative colitis (UC)) [2], significant weight loss (defined as severe malnutrition requiring preoperative total parental nutrition (TPN) for 7 days or more before first surgery), disease location and behaviour according to Montreal classification [8] (both of them confirmed at surgery), number and type of abdominal surgical recurrence/s, history of perianal fistula/s or abscess, number of surgeries and days of hospitalization, medical treatments with mesalamine (5-ASA), systemic corticosteroids, azathioprine, methotrexate and anti-TNFα therapy for more than 6 months before first surgery. Surgical recurrence was defined as a symptomatic clinical recurrence of CD, instrumentally diagnosed, not responding to medications, requiring surgery after a multidisciplinary decision as above described, macroscopically confirmed at the operation.

## Statistical analysis

In this study, all variables were categorical, except for age at diagnosis and days of hospitalization. Categorical data were presented as frequencies and percentages and the χ2 test was used to assess differences. The Kruskal–Wallis test was used to compare continuous variables, presented as median and interquartile range (IQR).

Logistic regression and survival models (time to recurrence) were calculated to estimate the probability of abdominal recurrence. Multivariable logistic regression analysis was performed using backward selection (*P*_out_ > 0.05), including all significantly associated variables from the univariate analyses. The results were reported as odds ratio (OR) with 95% confidence interval (CI). Comparisons among time of first recurrence curves were performed by log-rank (Mantel-Cox) test. A *p*-value of < 0.05 was considered statistically significant. All statistical analyses were conducted using Statgraphics Centurion XVI software and Graph-Pad Prism 7.00.

## Results

### Demographic and clinical characteristics of the study subjects

Out of 1283 patients initially identified in our database, 232 (18%) did not answer to the telephonic follow-up interview and were excluded. Therefore, 1051 CD patients were analysed. Their median age at first surgery was 37 years (IQR 29–48). The median follow-up time was 232 months (range 93–407), and specifically, 255 months (171–311 IQR) for G1, 254 (178–311) for G2, 201 (141–286) for G3 and 186 (124–286) for G4. In addition, the median follow-up time did not vary significantly among groups (*p* = 0.0957 by Kruskal–Wallis test). In total, 294 patients experienced surgical recurrences, and 158 (53.74%) a single abdominal recurrence, 69 (23.47%) two recurrences, 27 (9.18%) three and 40 (13.60%) four or more recurrences.

In Table 1, baseline clinical features of patients at their first surgery are shown.

**Table 1:** Clinical characteristics of patients, according to the age at first surgery

Age at the first surgery (years)	< 19 (G1)	20–39 (G2)	40–59 (G3)	> 60 (G4)	*P*-value
*n* (%)	46 (4.47)	544 (51.8)	368 (35.0)	93 (8.8)	
Age at first surgery, median years (IQR)	17 (15–18)	31 (26–35)	48 (43–53)	65 (62–69)	
Age at diagnosis, median years (IQR)	13 (10–16)	23 (19–28)#	40 (31–46)#	58 (49–64)#	0.0001
Time from diagnosis to abdominal surgery, median years (IQR)	3 (1–6)	5 (2–10)	7 (2–16)*	9 (2–18)°	0.0001
Male, *n* (%)	33 (71.7)	287 (52.7)	185 (50.3)	49 (52.7)	NS
Smoking status at first surgery, *n* (%)					
Current	1 (2.17)	206 (37.9)	96 (26.1)	25 (26.7)	0.0001
Never	45 (97.83)	285 (52.4)	220 (59.8)	43 (46.7)	
Former	0	12 (9.7)	52 (14.1)	25 (26.9)	
Family history of IBD, *n* (%)	1 (2.2)	10 (1.8)	9 (2.4)	3 (3.2)	NS
Disease location, *n* (%)					
L1 (ileal)	30 (65.2)	238 (43.8)	164 (44.6)	40 (43.0)	0.0083
L2 (colonic)	2 (4.3)	55 (10.1)	39 (10.6)	17 (18.3)	
L3 (ileo-colonic)	13 (28.3)	221 (40.6)	137 (37.2)	36 (38.7)	
L4 (isolated upper disease)	1 (2.2)	30 (5.5)	28 (7.6)	0	
Disease behaviour, *n* (%)					
B1 inflammatory	15 (32.6)	124 (22.8)	61 (16.6)	21 (22.6)	0.0013
B2 stricturing	20 (43.5)	287 (52.8)	232 (63.0)	62 (66.7)	
B3 penetrating	11 (23.9)	133 (24.4)	75 (20.4)	10 (10.8)	
*P* (concomitant perianal disease), *n* (%)	4 (8.7)	52 (9.6)	41 (11.1)	6 (6.5)	NS
History of perianal abscess, *n* (%)	17 (36.9)	168 (30.9)	87 (23.6)	16 (17.2)	0.0049
History of perianal fistula, *n* (%)	27 (58.7)	278 (51.1)	171 (46.5)	25 (26.9)	0.001
Severe malnutrition requiring preoperative TPN before first surgery, *n* (%)	4 (8.7)	12 (2.2)	6 (1.6)	2 (2.1)	0.0267
Previous long-term (> 3 months) medical treatment before first surgery, *n* (%)					
Steroids	7 (15.2)	124 (22.8)	83 (22.5)	24 (25.8)	NS
Anti-TNFα drugs	2 (4.3)	9 (1.6)	16 (4.3)	5 (5.4)	NS
Immunomodulators	2 (4.3)	20 (3.7)	17 (4.6)	6 (6.4)	NS
5-aminosalicylic acid	4 (8.7)	107 (19.7)	76 (20.6)	27 (29.0)	0.0393
Type of surgery					
-Strictureplasty/ies	3 (6.5)	59 (10.8)	47 (12.8)	6 (6.35)	0.0002
-Ileocecal resection	39 (84.8)	393 (72.2)	245 (66.6)	81 (87.1)	
-Ileocecal resection + strictureplasty/ies	0	29 (5.3)	41 (11.1)	2 (2.2)	
-Other bowel resections	4 (8.7)	63 (11.6)	35 (9.5)	4 (4.3)	
Total length of resected bowel at first surgery, cm	24 (15–30)	28 (20–43)	30 (15–45)	40 (23.5–55)*	0.0142
Short-term postoperative complications, *n* (%)	3 (6.52%)	17 (3.13%)	9 (2.45%)	5 (5.38%)	NS
Days of hospitalization, median (IQR)	10 (6–18)	17 (9–26)	13 (7–22)	12 (7–17)	0.0389
Surgical recurrence, *n* (%)	14 (30.4)	174 (32.0)	91 (24.7)	15 (16.1)	0.005
Abdominal surgeries for recurrence, *n* (%)					0.0107
0 (no surgical recurrence)	32 (69.6)	370 (68.0)	277 (75.3)	78 (83.9)	
1	5 (10.9)	90 (16.5)	55 (14.9)	8 (8.6)	
≥ 2	9 (19.6)	84 (15.4)	36 (9.8)	7 (7.5)	
Type of abdominal recurrence, *n* (%)					
-Stricturing	8 (17.4)	159 (29.2)	105 (28.5)	29 (31.2)	NS
-Penetrating	17 (37.0)	179 (32.9)	111 (30.2)	25 (26.9)	NS
-Lack of therapeutic response	0	4 (0.7)	0	3 (3.2)	-
-Peritonitis	0	3 (0.4)	0	0	-
-Other	0	6 (1.1)	4 (1.1)	1 (1.01)	NS

*IQR*, interquartile range; *NS*, not significantly different among groups. *TPN*, total parenteral nutrition. #*p* < 0.0001, ° < 0.001 *0.01 vs G1

### Significant differences among the four groups

Patients were categorized according to the age at first surgery as previously described in the method section. The number of patients for each group was 46 (4.47%) in G1, 544 (51.8%) in G2, 368 (9.8%) in G3 and 93 (8.8%) in G4. The four groups of patients exhibited significant differences in age at diagnosis, disease location and behaviour, incidence of perianal lesions (abscesses and/or fistulae) during follow-up time, incidence of recurrence and number of operation/s for surgical recurrence. The time span between CD diagnosis and abdominal surgery was significantly higher in G3 and G4 patients compared to G patients (Table 1) but did not vary in relation to the different location of disease (*p* = 0.0744).

Moreover, both preoperative severe malnutrition requiring preoperative TPN and median duration of hospitalization were also significantly different among groups (Table 1). On the contrary, significant differences in the incidence of short-term postoperative complications, type of recurrence and presence of concomitant perianal disease were not observed (Table 1).

The incidence of short-term postoperative complications was low in all groups (3.14% of all cases), mostly due to anastomotic leakage (15 out of 34 patients with surgical complications, 44%) and abdominal bleeding (9 case, 26.5%) (Table 2). Perioperative mortality (5–30 days after surgery) was observed in 9 cases, 5 patients with age at surgery between 40 and 59 and 4 over 60 years old.

**Table 2:** Patient with surgical complications, according to the age at the first surgery

	< 19 (G1)	20–39 (G2)	40–59 (G3)	> 60 (G4)
*n*	3	17	9	5
Abdominal bleeding requiring transfusion	1 (33.3%)	5 (29.4%)	2 (22.2%)	1 (20%)
Anastomotic leakage	1 (33.3%)	9(52.9%)	4 (44.4%)	1 (20%)
Thromboembolism	1 (33.3%)			
Abdominal hematoma		2 (11.8%)		
Abdominal abscess		1 (5.9%)	1 (11.1%)	2 (40%)
Abdominal bleeding requiring surgery			1 (11.1%)	
Bowel obstruction requiring surgery			1 (11.1%)	1 (20%)

At univariate logistic regression, age at surgery, age at diagnosis, disease location, history of perianal fistula or abscesses and previous long-term use of steroids, anti-TNFα or mesalamine were found significantly associated with recurrence risk. On the contrary, smoking does not seem to influence the risk of surgical recurrence; however, most smokers stopped smoking during the follow-up and this may have reduced the relevance of this variable.

**Fig. 1:**
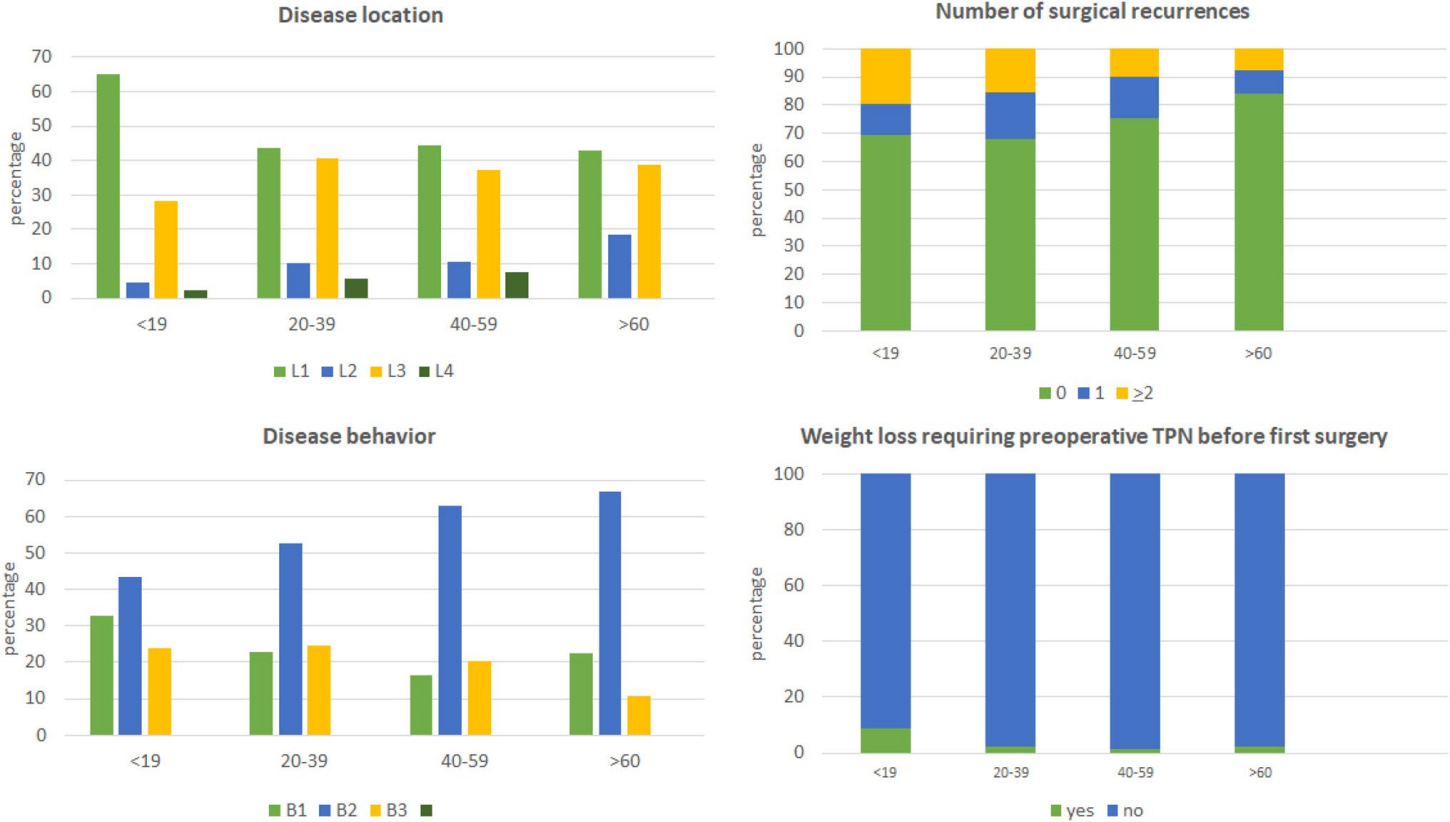
Disease location (**A**) and behaviour (**B**), number of patients with no recurrences, with one or 2 or more recurrences (**C**) and percentage of patients with weight loss before the first operation (**D**), according to the age at first surgery

Overall, younger patients had a significantly higher frequency of L1 and B1 disease (according to Montreal classification [8]) and exhibited more frequently weight loss and surgical recurrences (Fig. 1). On the contrary, G4 patients showed a higher incidence of L2 and L3 locations than the other groups, but only in 5 of them a rectal involvement was present (9.4%): rectal involvement was not statistically associated to the age at surgery.

**Fig. 2:**
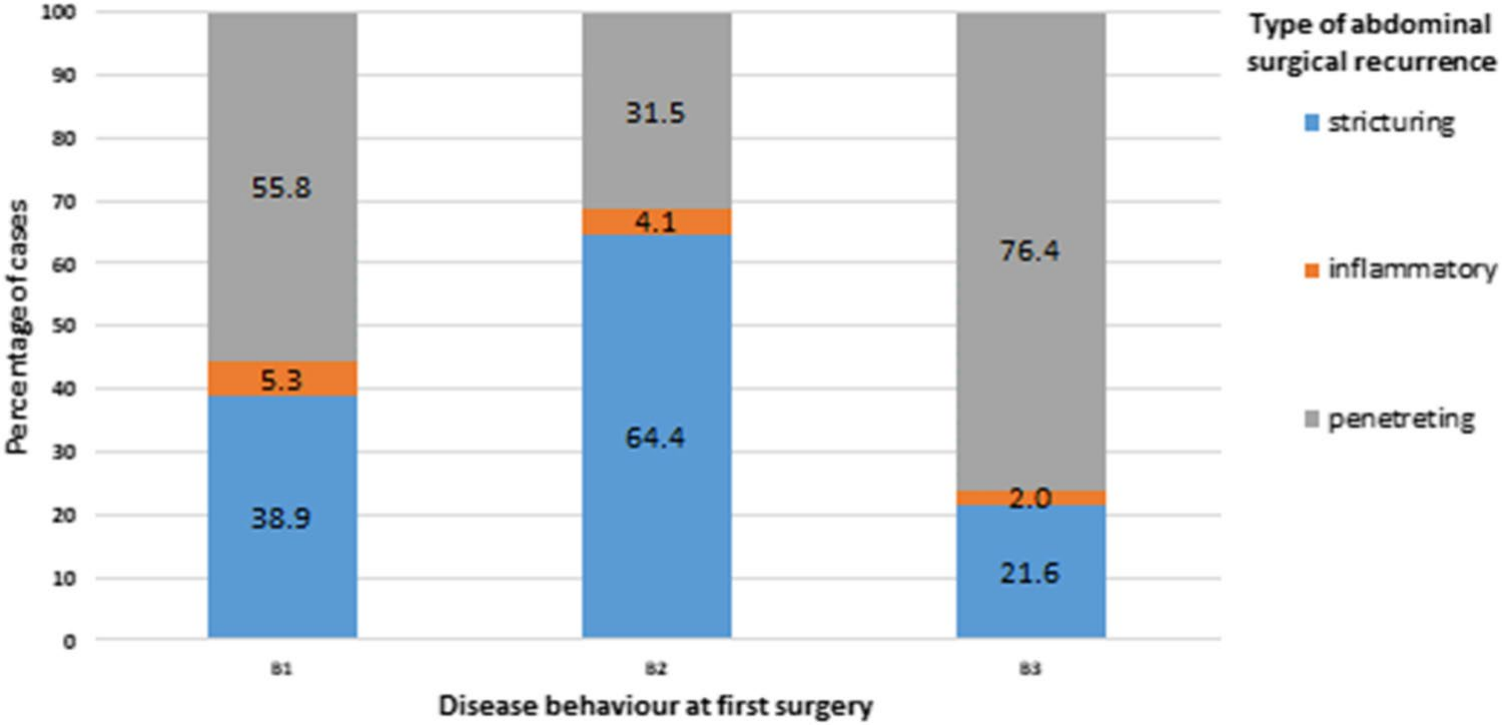
Association between disease behaviours and type of first abdominal surgical recurrence

Interestingly, the phenotype of surgical recurrence correlated with disease behaviour, specifically stricturing disease (B2) recurring mainly as stricturing disease (64.4% of the cases) and penetrating disease (B3) recurring as penetrating disease in most of the patients (76.4%, *p* < 0.0001), as shown in Fig. 2.

In multivariable logistic regression analysis, age at first surgery and L2 disease (OR 2.159, 95% CI: 0.049–1.49) was the two only independent risk factors for surgical recurrence (Table 3).

**Table 3:** Univariate and multivariable logistic regression analyses of the risk factors associated with surgical recurrence

Variable	Univariate model			Multivariate model		
	OR	95% CI	*p*-value	OR	95% CI	*p*-value
Age at surgery			0.0036			0.0000
G1	2.27	0.985–5.251		1.676	0.543–5.167	
G2	2.44	1.367–4.373		2.299	1.054–5.012	
G3	1.71	0.936–3.006		1.126	0.494–2.563	
Gender, male	0.890	0.680–1.166	0.399			
Age at diagnosis			0.0012			
A1	1.71	0.865–3.379				
A2	2.29	1.444–3.650				
Disease location			0.0000			0.0009
L1	0.480	0.252–0.915		0.418	0.111–1.569	
L2	1.854	0.912–3.769		1.902	0.497–7.281	
L3	0.820	0.434–1.549		0.895	0.247–3.244	
Disease behaviour						
B1	1.039	0.659–1.634	0.986			
B2	1.015	0.700–1.472				
No history of perianal fistula	0.740	0.563–0.973	0.031			
No history of perianal abscess	1.111	0.819–1.508	0.494			
Smoke habit			0.557			
No smokers	1.381	0.566–3.369				
Smokers	1.568	0.673–3.655				
No previous long-term use of steroids	0.564	0.415–0.766	0.0003			
No previous long-term use of anti-TNFa	3.864	1.169–12.774	0.0085			
No previous long-term use of ASA	0.59	0.429–0.811	0.0014			

Long-term: > 6 months, before first surgery

Moreover, the preoperative use of mesalamine was significantly higher in older patients while postoperative medical therapy was similar across groups.

### Association between abdominal CD recurrences at follow-up time with age

A separate analysis was conducted on patients operated before and after the age of 40 years finding heterogeneity regarding smoking habit, disease behaviour, history of perianal disease, surgical recurrence and previous treatment with anti-TNFα drugs (Table 4).

**Table 4:** Statistically different clinical characteristic in cumulative groups of patients below or over 40 years of age at first surgery

	G1 + G2	G3 + G4	*P*-value
No. patients	590	461	
Age at surgery, median years (IQR)	30 (24–35)	50 (44–57)	
Smoking status at first surgery, *n* (%)			0.0001
Current	207 (35.1)	121 (26.2)	
Never	330 (55.9)	263 (57.0)	
Former	12 (2.0)	77 (16.7)	
Disease behaviour, *n* (%)			0.0007
B1 inflammatory	139 (23.6)	82 (17.8)	
B2 stricturing	307 (52.0)	294 (63.8)	
B3 penetrating	144 (24.4)	85 (18.4)	
Type of surgery, *n* (%)			0.0189
-Strictureplasty/ies	62 (10.5)	53 (11.5)	
-Ileocecal resection	432 (73.2)	326 (70.7)	
-Ileocecal resection + strictureplasty/ies	29 (4.9)	43 (9.3)	
-Other resections	67 (11.4)	39 (8.5)	
History of perianal abscess, *n* (%)	185 (31.4)	103 (22.3)	0.0013
History of perianal fistula, *n* (%)	305 (51.7)	196 (42.5)	0.0034
Surgical recurrence, *n* (%)	188 (31.9)	106 (23.0)	0.0015
Surgeries for recurrence, *n* (%)			0.0023
0	402 (68.1)	355 (77.0)	
1	95 (16.1)	63 (13.7)	
≥ 2	93 (15.8)	43 (9.3)	
Long-term treatment with anti-TNFα drugs before first surgery, *n* (%)	11 (1.9)	21 (4.6)	0.0174
Permanent end ileostomy	2 (0.34%)	6 (1.30%)	0.1482

*IQR* interquartile range. Long-term: > 6 months, before first surgery

**Fig. 3:**
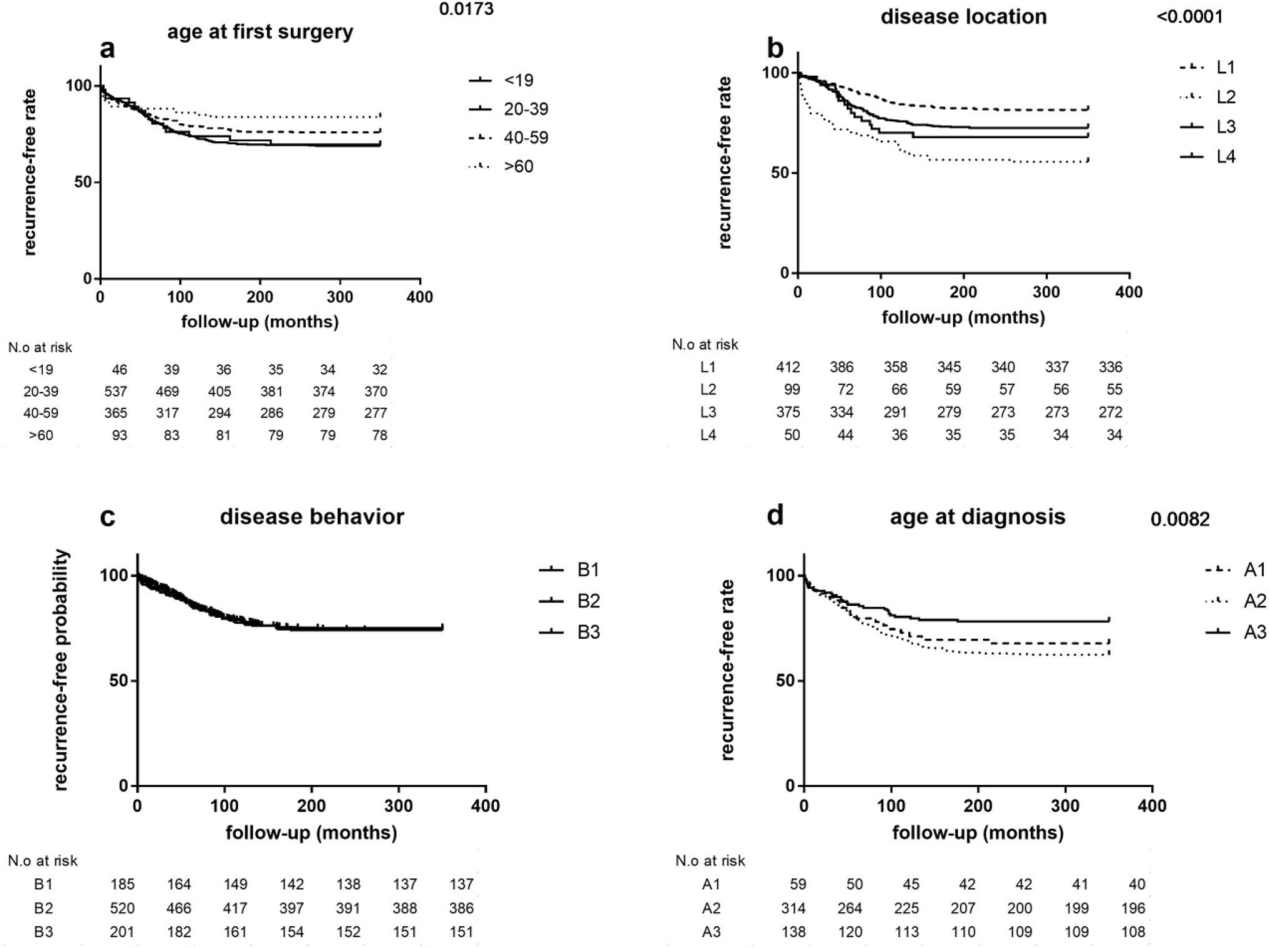
Recurrence-free probability curves for CD patients according to age at first surgery (**a**), age at diagnosis (**b**), disease behaviour (**c**) and disease location (**d**), by log-rank test

By log-rank (Mantel-Cox) test, the rate of patients with abdominal CD recurrence at follow-up time was significantly associated with younger age (both at diagnosis and at the first surgery) and L2 location. On the other hand, patients ≥ 60 years old at first surgery or with L1 disease had the highest recurrence-free time (Fig. 3).

## Discussion

Our experience highlighted statistically significant differences among the four groups analysed, in detail, age at diagnosis, smoke habit, time between diagnosis and surgery, disease location and behaviour, history of perianal fistula or abscess, severe malnutrition requiring total parental nutrition before surgery, type of surgery, total length of resected bowel, median duration of hospitalization, incidence of abdominal recurrences and number of surgical recurrences.

Moreover, the group 1 displays an inverse linear trend with time in the severity of clinical characteristics when compared to group 4. On the contrary, the incidence of short-term complications, types of abdominal recurrence and presence of concomitant perianal disease did not vary among groups. The age at surgery and the disease location were the only independent risk factors for abdominal surgical recurrence at multivariate analysis.

Many data are already present in the literature about the relation between age of onset and CD behaviour [4, 6, 9]. However, the peculiarity of our manuscript is to describe a cohort of operated CD patients, prospectively followed after surgery for a long-term period (≥ 7 years), focusing on the age at first surgery, rather than age at diagnosis. In our opinion and from a surgical point of view, this approach may be relevant in terms of expected outcomes and can minimize biases due to the previous CD duration, diagnostic delay and medical therapies, which often provide heterogeneous results among patients. It is well-known that both resection and strictureplasty performed in high-volume centres are able to control CD complications and its symptoms. In this scenario, the first operation can represent a new starting point for the clinical history of every CD patient undergoing surgery. Moreover, postoperative recurrence is the most frightening event for patients affected by CD, and it is well-known it can be present, in a clinical or subclinical manner, already 6–12 months after surgery [10]. For all these mentioned reasons, we chose to analyse our wide cohort characteristics in relation to the age at the first operation, evaluating the influence of specific clinical features in the different groups. And for the same reasons, we focused on surgical recurrence, in order to minimize the bias connected with the definition of clinical recurrence of CD in our long-lasting observational period. In fact, we believe surgical recurrence is the only unconfutable indicator of a symptomatic CD recurrence unresponsive to medical therapies, macroscopically defined during the surgical operation, and microscopically confirmed after specimen pathological analysis.

Smoking habit is maybe the most analysed risk factor for medical and surgical recurrences [11, 12]. In our cohort, only 26% of elderly (G4, ≥ 60 years) and patients between 40 and 59 years (G3) were active smokers vs 37.9% in G2 (*p* = 0.0001). It is to be noted that more than 50% of the surgical cohort of patients had never smoked (56.4%) and at the end of follow-up, the majority of smokers reported to have quit smoking. Although smoking is a known risk factor for recurrences in CD, curiously, at univariate analysis, smoke habit did not emerge as a risk factor for surgical recurrence in our cohort (Table 3). Regarding the disease location, we found statistically significant differences within the four age-related groups, with ileal location (L1) being more frequent in younger patients, while colonic involvement (L2) increased with age. This is consistent with previous data reporting that patients diagnosed with CD after the age of 50 were more likely to have isolated colonic disease and inflammatory disease behaviour, with less penetrating complications or perianal disease [5]. About CD behaviour, we observed a statistically significant increase in stricturing and a reduction in penetrating diseases with age, from 43.5 (G1) to 66% (G4) and from 23.9 (G1) to 10.8% (G4), respectively. Interestingly, if we correlate this data with the different surgical strategy that was performed in our cohort (strictureplasty/ies alone, bowel resection/s alone, resection plus strictureplasty/ies), we may postulate that G4 patients are characterized by a less aggressive disease at first surgery. In fact, G4 patients were found to undergo resection alone more frequently than the other age groups. On the other hand, it is well-known that resection is destined for macroscopically short disease, usually of the terminal ileum, or in presence of fistula/abdominal abscess where strictureplasty is contraindicated. Therefore, this cross relation confirms a poorly described finding, which is the presence of a peculiar disease for G4 patients, often characterized by a short disease (usually at the terminal ileum) and with a less aggressive behaviour. However, regarding the total length of resected bowel at first surgery, we found that the elderly underwent relatively longer resections than G1 patients. This could be related to the surgeon perception of the patient’s disease according to the age at first surgery, preferring to preserve as much bowel as possible when the first abdominal CD operation is performed before the age of 20. The association with perianal disease was homogeneous among the four groups, although patients operated after 60 years (G4) were the ones with the lowest correlation (6.5%).

When dividing the cohort into two separate groups of patients < 40 and > 40 years, a statistically higher history of perianal disease was reported in younger patients, consistent with data already reported in literature that show a higher prevalence and cumulative incidence of perianal disease in younger adults [13]. In fact, we found a significantly lower prevalence of abscesses and fistulas in elderly group. This result could appear contradictory as G4 patients suffered the highest percentage of colonic location, but it is to notice that about the 17 G4-L2 patients, a rectal involvement was found in only 5 of them.

In addition, the same decreasing trend (a linear decrease from G1 to G4 groups) described about perianal involvement was observed in both surgical recurrence incidence and in the need for recurrent surgeries. This may be the consequence of differences among cohort groups as older patients showed a lower prevalence of isolated small bowel disease and penetrating involvement, factors associated with a higher risk of Crohn’s recurrence over time [14]. In this regard, our data are consistent with another report showing a rarer occurrence of recurrence in elderly than in younger patients (43% vs 64%) [15], despite other conflicting results showing a similar or higher recurrence in the G4 group [16, 17].

From the opposite point of view, patients in G1 group underwent a lower period of medical treatments before the first surgery, probably due to a top-down approach or to a more aggressive disease behaviour that was less responsive to medical therapies. Few other studies have addressed this topic, demonstrating that the need for surgery was indeed lower in patients with elderly onset of the disease [18, 19].

Accordingly, G1 patients had significantly higher frequency of severe malnutrition requiring TPN than G2-G4, highlighting a more aggressive systemic inflammation, directed towards catabolism. Interestingly, the catabolic status was not related to the length of the hospital stay, as G1 patients experienced a significantly shorter postoperative course. Surprisingly, G4 and G3 patients showed shorter stay than G2 group, despite previous data suggested worse outcomes, longer in-hospital stay, and higher mortality rate after surgery for elderly IBD patients [20, 21]. Noteworthy, our aim was not to evaluate frail patients, as specific comorbidities were not taken into account. Moreover, the length of the overall mean hospital stay is probably biassed by the long-term observational period of the study, indeed the ERAS (enhanced recovery after surgery) protocols, introduced to reduce the postoperative stay, have been adopted at our centre starting from 2016, and primary laparoscopic approach in each patient since 2013. The long mean follow-up period (232 months) with different approaches across the decades may be seen as a weak point, but we believe that the monocentric setting and the large sample size of our cohort are two important strength points to reach our aim.

Furthermore, the prophylactic protocols with monoclonal antibodies changed with time, and this could be a limitation of the present study; however, the fact that the mean follow-up is not significantly different in G1-G4 groups limits the bias connected to the length of our observational period.

To the best of our knowledge, evidence about long-term clinical behaviour of CD according to the age at first abdominal CD surgery are lacking in the international literature.

In conclusion, elderly CD patients show clinical differences and peculiarities when related to other age groups of patients: a linear trend was found in many analysed clinical variables, with a more indolent course in patients undergoing their first surgery in elderly, both in terms of disease behaviour and occurrence of surgical recurrence. On the contrary, G1 patients seem to be characterized by a more aggressive disease course. These aspects should be considered and shared with the patient and gastroenterologists when surgery is indicated, starting from the first abdominal operation, in order to perform a tailored surgical strategy, and even in approaching the follow-up. An accurate long-term follow-up should be recommended and all the efforts should be made by clinicians to adequately treat patients requiring first abdominal surgery at young age.

## Abbreviations

CD: Crohn’s disease
IBD: Inflammatory bowel disease
5-ASA: Mesalamine
IQR: Interquartile range
TNFα: Tumour necrosis factor alfa
OR: Odds ratio (OR)
CI: Confidence interval
ERAS: Enhanced recovery after surgery
